# Evaluating Yoga-Based Intervention Versus the American Diabetes Association Exercise Regimen in Conjunction With Standard Care for Autonomic Neuropathy in Diabetes Mellitus: An Exploratory Clinical Trial

**DOI:** 10.7759/cureus.61329

**Published:** 2024-05-29

**Authors:** Ramesh Kumar, Puneet Dhamija, Gyan Vardhan, Ravi Kant, Yogesh Singh, Raj Kumar Yadav, Bhandari Rudra, Monika Pathania

**Affiliations:** 1 Pharmacology, All India Institute of Medical Sciences, Rishikesh, IND; 2 General Medicine, All India Institute of Medical Sciences, Rishikesh, IND; 3 College of Nursing, All India Institute of Medical Sciences, Rishikesh, IND; 4 Physiology, All India Institute of Medical Sciences, Rishikesh, IND; 5 Physical Medicine and Rehabilitation, All India Institute of Medical Sciences, Rishikesh, IND; 6 Science of Yoga, University of Patanjali, Haridwar, IND; 7 Internal Medicine, All India Institute of Medical Sciences, Rishikesh, IND

**Keywords:** nerve conduction velocity, depression, quality of life, diabetic autonomic neuropathy, diabetes mellitus, adae, yoga-based intervention

## Abstract

Introduction: Diabetic autonomic neuropathy (DAN) is a prevalent yet often overlooked complication of diabetes mellitus (DM), impacting multiple organs and substantially elevating the risk of morbidity and mortality. This study aimed to assess the effectiveness of yoga-based intervention (YBI) compared to the American Diabetes Association exercise regimen (ADA Ex. Regime) and standard care for treating autonomic neuropathy in type 2 DM.

Methods: This open-label exploratory clinical trial featured two parallel study arms: Group A (Intervention), which received YBI alongside standard care, and Group B, which adhered to the ADA Ex. Regime in conjunction with standard care. A total of 80 participants aged 35-60, diagnosed with type 2 DM and autonomic neuropathy, were equally allocated to both groups. Data collection included nerve conduction velocity (NCV) tests, autonomic function tests (AFTs), as well as evaluations of depression and quality of life.

Results: YBI demonstrated a drop in parasympathetic tone compared to the ADA Ex. Regime. Following a six-month intervention, the sympathetic activity indicator (SD2) exhibited a significantly lower value in the YBI group than in the ADA Ex. Regime group, indicating a positive effect (p < 0.05), while the ADA Ex. Regime showed more improvement in certain areas of NCV (e.g., left and right peroneal NCV, right and left peroneal F-latency), notable differences were observed in alkaline phosphatase levels, depression scores, and WHO-5 wellness, all reaching statistical significance at p < 0.05.

Conclusions: The study findings observed that a 24-week YBI significantly reduced in symptoms of diabetic neuropathy and stress. Although the ADA Ex. Regime demonstrated greater improvement in specific aspects of NCV compared to YBI, YBI outperformed the ADA Ex. Regime in enhancing WHO-5 wellness and reducing depression symptoms.

## Introduction

Diabetic autonomic neuropathy (DAN) is a common yet relatively unknown complication of diabetes mellitus (DM) [1,2]. DAN can result in the dysfunction of one or multiple organ systems regulated by the autonomic nervous system (ANS), or it can affect the entire ANS [3]. The prevalence of DAN is uncertain due to variations in the diagnostic criteria used [4]. It affects various organs and significantly increases the risk of illness or death and mortality rates [5,6]. Sensory symptoms, such as pain, numbness, tingling, pins and needles, and prickling, are associated with DAN [7]. It is a relatively obscure complication. Despite its significant negative influences, the survival and quality of life of individuals with diabetes. There is a paradoxical association between inadequate glucose control and prompt treatment of high blood sugar levels, which can contribute to an elevated risk of neuropathy. Glycaemic control refers to a reduction of more than 2% points in glycosylated HbA1c levels for three months [8]. Yoga, as a complementary therapy, has an extensive historical record of being utilized to manage type 2 diabetes mellitus (T2DM) in India [9]. Yoga activates the parasympathetic nervous system, promoting the restoration of a healthy balance in the ANS. When our body or mind detects a threat or experiences stress, regardless of whether it is considered "positive" or "negative" stress, the sympathetic nervous system, also referred to as our "emergency response system," is activated [10]. Yoga techniques have the potential to reduce blood pressure and pulse rate, facilitate breathing, and enhance heart rate variability, all of which are positive indicators of good health and improved parasympathetic tone [11]. Medical yoga/applying yoga incorporates essential breathing techniques, mindfulness, and other elements alongside its physical aspects, which are vital and beneficial for enhancing bodily strength. Practicing regular meditation and dedicating time to introspection and study to maximize the benefits [12]. However, resistance exercise has also been associated with insulin sensitivity. Factors to consider include sensitivity, daily energy consumption, and quality of life [13,14]. This study was conducted to evaluate the effect of yoga-based intervention (YBI) and American Diabetes Association (ADA)-recommended exercise regimen (ADA Ex. Regime) on autonomic function in terms of change in biochemical parameters, autonomic function test (AFT), nerve conduction velocity (NCV) of lower limbs, psychological well-being among autonomic neuropathy in T2DM patients.

## Materials and methods

Study design

**Figure 1:**
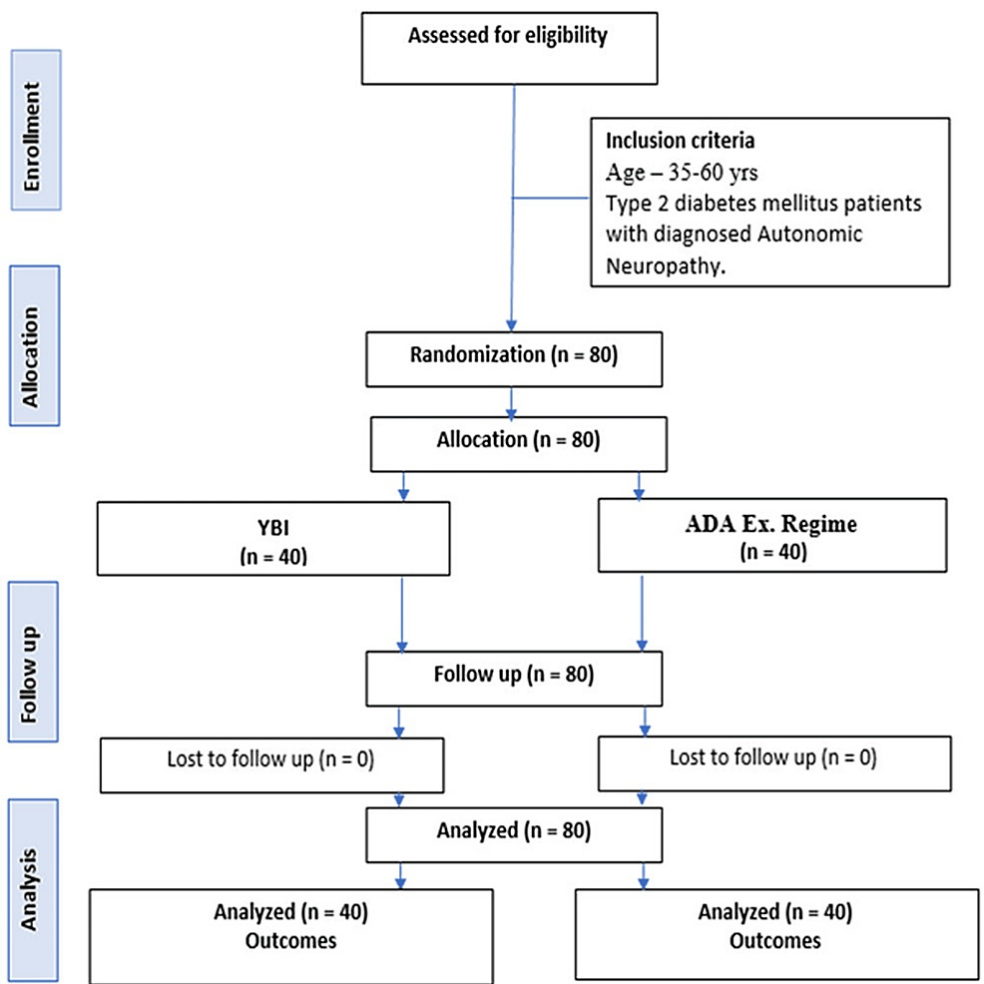
Study flowchart

A cohort of 80 participants, evenly distributed between two groups, was chosen from the General Medicine Outpatient Department. After written consent, participants were equally distributed to either Group A (Intervention Group) or Group B (ADA Ex. Regime Group) for all eligible patients. The research spanned 24 months, from December 2021 to January 2024. As illustrated in Figure 1, all patients were followed up after baseline and six months for all the outcomes.

T2DM-diagnosed patients aged 35-60 with autonomic neuropathy and willing to participate were selected for inclusion after systemic examination and necessary investigations. Specific performance was used to collect the data.

Patients opting for treatment did not comply with inclusion criteria. Patients with exclusion criteria were those on insulin, those with HbA1c > 11%, and those with acute macrovascular complications, cancer, pulmonary tuberculosis, and rheumatoid arthritis. Also under the exclusion criteria were those who were unable to perform yoga and receiving drugs like drotaverine, beta-blockers, or any other drug likely to affect AFTs, as well as patients with a history of angina/uncontrolled hyperglycemia/any other contraindication applied by the ADA.

Intervention

Group A (YBI): The intervention involved a blend of yoga practices encompassing asanas, pranayama, kriya, mudra-bandh, and dhyan. The yoga instructor administered the intervention, initially training participants at the study center. Subsequently, participants were directed to engage in their specific techniques during five weekly online sessions lasting 60 minutes and conducted through Zoom. The exercise protocol was attached in Supplemental Material 1. A certified yoga trainer supervised sessions.

Group B (ADA Ex. Regime): ADA combines exercises and techniques provided by physiotherapists. Participants were instructed to practice their respective techniques for 60 minutes of online sessions five days in a week conducted through Zoom. The exercise protocol is in the Supplemental Material 2. A trained physiotherapist supervised the sessions.

Precautions taken during the sessions

Selected patients were asked to keep fast-acting glucose snacks to treat hypoglycemia if it develops during the exercise, avoid eating large meals before the session, avoid rapid changes in movement that may result in fainting, and avoid activities in environmental extremes. Extra fluids were allowed to be consumed to protect against dehydration and hyperthermia.

Outcome variables and data collection tools

Primary Outcome

The AFT was done for all selected patients. AFT was done at baseline and after six months follow-up by ECG in the AFT lab of the physiology department. Analysis of heart rate variability was done with the help of the PowerLab version (8.0) application through LabChart form. Patients were advised not to consume any tea, coffee, alcohol, or medications that may impact the nervous system for 24 hours before to the AFT. AFT data recording process was adopted [15,16].

Secondary Outcome

NCV: An electrical impulse's speed across a patient's lower limb was measured by an NCV test, also known as a nerve conduction study (NCS). The selected patient’s nerve is stimulated throughout the test, typically using electrode patches affixed to the skin of lower limbs [17]. The data recording process was adopted as per the guidelines of the Department of Physiology at AIIMS Rishikesh.

Blood sample collection: 4 mL of blood sample was collected in plain vial and serum was separated for biochemical analysis (lipid profile, liver function test and renal function test) in fasting and again 2 mL of blood sample was collected two hours post breakfast for postprandial blood sugar (PPBS).

Psychological well-being test: Psychological tests, WHO-5 Well-Being Index (WHO-5) and patient depression inventory PHQ-9, were administered in both groups of patients at baseline and after six months follow up. Both of tests were paper-pencil tests. The WHO Regional Office in Europe initially launched the measure in its current format in 1998 as a component of the DEPCARE project on well-being measures in primary healthcare [18]. All selected patients were advised to read the test instructions carefully and tick the right option after choosing the appropriate answer according to their situation. Patients were requested to complete both tests in 15-20 minutes. The WHO-5 is a concise, self-reported measure of current mental health, while the PHQ-9 is used to monitor and assess the severity of depression over time in newly diagnosed patients or those undergoing treatment for depression [19,20].

Statistical analysis

The statistical presentation includes the mean with SD, median with interquartile range (IQR), and various clinical features of the patients. The analysis utilized Statistical Package for Social Sciences (SPSS Inc, Chicago, IL, version 23.0 for Windows) and Microsoft Excel 2010. Mean ± SD was used to express quantitative data, while qualitative or categorical variables were depicted as proportions. The normality of quantitative data was assessed using the Shapiro-Wilk test (P < 0.05). The Wilcoxon signed-rank test compared 'after six-month' values with 'baseline' values between the groups. Additionally, multiple linear and logistic regression analyses were applied, as applicable, to evaluate the effects of covariates. A p-value of 0.05 or lower was considered statistically significant.

## Results

This study was conducted as an open-label exploratory clinical trial from December 2021 to January 2024. It employed a parallel two-arm design, comprising two distinct study groups illustrated in Figure 1. Group A (Intervention) involved a YBI combined with standard care, while Group B involved adherence to the ADA Ex. Regime alongside standard care. The primary objective was to assess and measure the outcomes of these interventions. All patients in both group were enrolled in study according to inclusion and exclusion criteria. Patients were enrolled after written consent approved by Institutional Ethics Committee and trial was registered at CTRI following number is: CTRI/2021/12/039068. The complete follow-up and analysis of all participants further strengthen the validity of the results.

Demographic characteristics

The demographic characteristics (Table 1) compare the two groups, YBI and ADA, across several parameters using descriptive statistics and independent t-tests to identify any significant differences. The analysis shows that the mean ages of the YBI (52.5 years) and ADA (51.33 years) groups are similar, with no significant difference (p = 0.71). The YBI group has a mean height of 165.85 cm, significantly taller than the ADA group’s mean height of 163.02 cm (p = 0.03). The YBI group averages 70.9 kg for weight, while the ADA group averages 67.94 kg, but this difference is insignificant (p = 0.09). The BMI values are close, with means of 25.70 kg/m² for YBI and 25.47 kg/m² for ADA, showing no significant difference (p = 0.65). The duration of DM is also similar, with means of 90.8 months for YBI and 110.46 months for ADA (p = 0.93). Lastly, the hypertension duration (HT) is nearly identical, with YBI at 48 months and ADA at 48.66 months, with no significant difference (p = 0.11). Overall, only the height parameter shows a statistically significant difference between the two groups, while other parameters do not. Other demographic characteristics such as gender, family history, education and occupation are described in Table 2. 

**Table 1:** Demographic characteristics of both groups (YBI/ADA Ex. Regime) n=80 (40:40) YBI: Yoga-based intervention; ADA Ex. Regime: American Diabetes Association exercise regimen; DM: Diabetes mellitus; HT: Hypertension duration

Parameter	Groups	N	Minimum	Maximum	Mean±SD	t-value	p-value
Age (years)	YBI	40	35	60	52.5±7.17	0.37	0.71
	ADA Ex. Regime	40	36	60	51.33±11.88		
Height (cm)	YBI	40	153	176	165.85±6.40	2.15	0.03
	ADA Ex. Regime	40	154	172	163.02±26.41		
Weight (kg)	YBI	40	51	97	70.9±10.21	1.69	0.09
	ADA Ex. Regime	40	57	77	67.94±11.76		
BMI (kg/m^2^)	YBI	40	19	35	25.70±3.59	0.44	0.65
	ADA Ex. Regime	40	21.5	28.7	25.47±4.29		
DM (months)	YBI	40	24	252	90.8±64.11	0.58	0.93
	ADA Ex. Regime	40	36	252	110.46 61.27		
HT (months)	YBI	16	12	144	48±30.03	1.13	0.11
	ADA Ex. Regime	18	24	144	48.66±33.04		

**Table 2:** Participant's demographic characteristics YBI: Yoga-based intervention; ADA Ex. Regime: American Diabetes Association exercise regimen

Attributes		Groups	
		YBI (N=40)	ADA Ex. Regime (N=40)
Gender	Male	29	26
	Female	11	14
Family History	Yes	12	20
	No	16	12
	Not Known	12	8
Education	Primary	1	3
	Matriculation	7	9
	Intermediate	10	14
	Graduation	13	10
	Postgraduation	9	3
Occupation	Advocate	0	2
	Carpenter	1	2
	Farmer	9	5
	Housewife	10	13
	Manager	2	1
	Nurse	1	1
	Policeman	0	1
	Retired	3	3
	Shopkeeper	9	9
	Teacher	5	3

​​​​Impact of YBI and ADA Ex. Regime on AFT

Table 3 presents the mean and SD changes between a YBI and an ADA Ex. Regime on autonomic functions in patients with diabetic neuropathy after six months of the intervention period. The analysis reveals several noteworthy findings across various parameters. The average RR interval slightly decreased in the YIB group but increased in the ADA Ex. Regime group, with no statistically significant difference observed (p = 0.227). Both groups showed a slight increase in the median RR interval, with no significant difference between them (p = 0.361). YIB led to a significant decrease in SDRR (p = 0.010), indicating improved heart rate variability. However, there was no significant change in the ADA Ex. Regime group. The coefficient of variation of R-R intervals (CVRR) significantly decreased in the YIB group (p = 0.009), suggesting enhanced heart rate variability, while no significant change was observed in the ADA Ex. Regime group. YIB demonstrated a significant decrease in heart rate (p = 0.002), indicating improved heart rate variability, whereas no significant change was observed in the ADA Ex. Regime group. Both SD of successive differences (SDSD) and root mean square of successive differences (RMSSD) significantly decreased in the YIB group (p = 0.014), indicating improved heart rate variability. No significant changes were observed in the ADA Ex. Regime group. Although there was a non-significant decrease in pRR50 in the YIB group, a slight increase was noted in the ADA Ex. Regime group. Total power decreased in both groups without statistical significance. However, YIB showed a significant decrease in very low frequency (VLF) power (p = 0.022), indicating improved autonomic function. The two groups had no significant changes in low frequency (LF) and high frequency (HF) powers. While the LF/HF ratio decreased in the ADA Ex. Regime group (p = 0.146). YIB led to a significant increase in the Valsalva maneuver index (p = 0.048), suggesting improved autonomic function, whereas a slight decrease was observed in the ADA Ex. Regime group. No significant changes in the latency to symptom threshold (LsT) ratio were observed in either group.

**Table 3:** AFTs : Impact of a YBI and ADA Ex. Regime in diabetic neuropathy patients over a six-month period, from baseline Non-Parametric Test: Comparison of two samples (Wilcoxon, Mann-Whitney test) compared the difference of post-data with pre-data in the YBI group and post-data with pre-data in the ADA Ex. Regime group. *Some of the parameter’s P values are significant at <0.05. SDRR (ms): SD of R-R intervals; CVRR: Coefficient of variation of R-R interval; SDSD: SD of the successive differences between adjacent N-Ns (normal-to-normal R-R intervals); RMSSD: Root mean square of successive differences (between heartbeats in milliseconds); pRR50: Proportion of NN50 divided by total numbers of NN; VLF power: Very low frequency; LF power: Low frequency power; HF power: High frequency power; SD1: SD of Poincaré plot perpendicular to the line of identity; SD2: SD of Poincaré plot along the line of identity; DBT: Deep breathing test; LsT ratio: Latency to symptom threshold ratio; YBI: Yoga-based intervention; ADA Ex. Regime: American Diabetes Association exercise regimen; IQR: Interquartile range

AFT parameters	Intervention	Inter group change difference in mean ± SD (at baseline)	Inter group change difference in mean ± SD (at six months)	P-value	Median	IQR
Average RR	YBI	713.53 ±227.42	-2.009 ± 292.383	0.227	-10.55	172.96
	ADA Ex. Regime	730.10 ±193.95	43.647 ± 286.152		22.43	178.47
Median RR	YBI	713.07 ±229.34	5.501 ± 300.682	0.361	-19.25	185.88
	ADA Ex. Regime	730.29 ±196.18	40.990 ± 286.606		25.15	189.73
SDRR (ms)	YBI	55.58 ±57.36	-24.254 ± 61.613	0.010	-13.62	38.14
	ADA Ex. Regime	38.83 ±39.10	-0.067 ± 48.245		6.09	22.92
CVRR	YBI	2.07 ±12.38	-2.035 ± 12.391	0.009	-0.01	0.04
	ADA Ex. Regime	0.05 ±0.05	-0.003 ± 0.069		0.01	0.03
Average Rate	YBI	78.24 ±11.93	-1.680 ± 15.364	0.170	0.74	21.24
	ADA Ex. Regime	78.54 ±11.32	-4.985 ± 13.627		-4.56	18.09
SD Rate	YBI	5.82 ±6.60	-2.862 ± 7.004	0.002	-1.19	2.90
	ADA Ex. Regime	4.03 ±4.63	-0.600 ± 5.162		0.11	2.39
SDSD	YBI	50.37 ±76.81	-24.568 ± 81.950	0.014	-6.41	25.19
	ADA Ex. Regime	32.31 ±52.67	-0.450 ± 65.531		4.67	24.87
RMSSD	YBI	51.74 ±77.00	-26.089 ± 82.205	0.014	-6.41	25.16
	ADA Ex. Regime	32.21 ±52.63	-0.485 ± 65.451		4.67	24.83
pRR50	YBI	13.79 ±23.12	-5.067 ± 24.283	0.118	-0.65	16.82
	ADA Ex. Regime	12.18 ±19.16	4.627 ± 19.992		1.97	9.30
Total Power (ms²)	YBI	3678.85 ±14284.49	-1729.761 ± 15005.454	0.188	-45.80	1998.23
	ADA Ex. Regime	3905.48 ±17552.52	-1894.102 ± 17995.859		516.35	1807.26
VLF Power (ms²)	YBI	5163.40 ±26833.06	-4696.284 ± 26869.336	0.022	-147.94	1239.27
	ADA Ex. Regime	883.91 ±2187.93	-215.264 ± 2362.760		187.20	764.85
VLF (%)	YBI	49.00 ±22.40	0.831 ± 28.991	0.900	4.48	40.73
	ADA Ex. Regime	47.89 ±22.50	1.396 ± 26.604		2.63	29.68
LF Power (ms²)	YBI	833.19 ±2564.71	-387.265 ± 2802.666	0.170	4.71	558.46
	ADA Ex. Regime	1078.92 ±4321.79	-584.900 ± 4423.925		90.22	452.74
LF (%)	YBI	24.35 ±11.30	0.885 ± 13.633	0.397	-0.07	19.34
	ADA Ex. Regime	25.01 ±12.63	-3.269 ± 13.760		-1.60	18.74
LF Power (nu)	YBI	51.77 ±21.30	3.624 ± 26.366	0.183	2.45	34.69
	ADA Ex. Regime	52.08 ±20.56	-3.570 ± 25.295		-2.69	31.02
HF Power (ms²)	YBI	1733.19 ±8315.21	-1031.652 ± 8654.652	0.162	-38.93	335.35
	ADA Ex. Regime	1692.15 ±9188.88	-932.461 ± 9428.330		142.50	684.13
HF (%)	YBI	24.95 ±18.15	-0.921 ± 22.462	0.347	-1.21	25.31
	ADA Ex. Regime	25.75 ±18.45	1.802 ± 20.602		1.40	19.03
HF Power (nu)	YBI	45.43 ±18.66	-2.070 ± 24.039	0.132	-0.21	33.4
	ADA Ex. Regime	45.18 ±18.34	4.876 ± 19.656		3.28	27.25
LF/HF Ratio	YBI	2.00 ±2.59	0.127 ± 3.093	0.146	0.08	2.26
	ADA Ex. Regime	1.76 ±1.88	-0.440 ± 2.104		-0.05	1.44
SD1	YBI	36.69 ±58.13	-18.371 ± 61.636	0.009	-4.53	17.8
	ADA Ex. Regime	22.78 ±37.22	-0.302 ± 45.456		2.65	18.52
SD2	YBI	67.57 ±62.91	-28.542 ± 66.651	0.008	-17.93	49.56
	ADA Ex. Regime	48.77 ±42.31	-0.145 ± 51.191		7.42	25.96
DBT	YBI	11.18 ±5.38	1.471 ± 8.701	0.092	0.91	9.24
	ADA Ex. Regime	11.08 ±7.66	-1.436 ± 9.268		-0.77	8.86
Valsalva	YBI	1.14 ±0.11	8.040 ± 24.664	0.048	0.06	0.21
	ADA Ex. Regime	1.15 ±0.11	-0.017 ± 0.173		-0.02	0.15
LsT Ratio	YBI	1.05 ±0.08	0.018 ± 0.197	0.806	0.02	0.18
	ADA Ex. Regime	1.02 ±0.66	-0.004 ± 0.072		0.00	0.10

The observation of this study suggests that the YBI had a more significant impact on improving certain autonomic functions, remarkably heart rate variability, compared to the ADA Ex. Regime.

Impact of YBI and ADA Ex. Regime on NCV

Table 4 presents the changes observed between a YBI and an ADA Ex. Regime on NCV in patients with diabetic neuropathy after a 6-month intervention period. Both groups showed a slight increase in peroneal nerve latency, with YBI demonstrating a non-significant decrease in left peroneal nerve latency (p = 0.058). ADA Ex. Regime group showed no significant changes. There were no significant changes in peroneal nerve amplitude in either group. The YBI led to a significant decrease in left peroneal nerve NCV (p = 0.016), whereas the ADA Ex. Regime group showed a significant increase in both right and left peroneal nerve NCV (p = 0.048 and p < 0.05), respectively. Both interventions showed slight increases in tibial nerve latency, with no significant differences between the groups and no significant changes in tibial nerve amplitude were observed in either group. The YBI group demonstrated no significant changes in tibial nerve NCV, while the ADA Ex. Regime group showed significant increases in both right and left tibial NCV (p < 0.05). YBI led to significant decreases in F-latency for both peroneal and tibial nerves (p = 0.014 and p = 0.036), respectively, while the ADA Ex. Regime group showed significant increases (p < 0.05). No significant changes in F-wave conduction velocity (FWCV) were observed in either group. Overall, the observation indicates differential effects of YBI and ADA Ex. Regime on NCV parameters. YBI showed improvements in certain parameters such as peroneal nerve latency and F-latency, while ADA Ex. Regime demonstrated increases in peroneal and tibial NCV. These findings suggest that each intervention may have distinct impacts on NCV in patients with diabetic neuropathy, highlighting the importance of tailored treatment approaches for this population.

**Table 4:** Impact of YBI and ADA Ex. Regime on NCV Non-Parametric Test: Comparison of two samples (Wilcoxon, Mann-Whitney test) compared the difference of post data with pre-data in the YBI group and the difference of post data with pre-data in the ADA Ex. Regime group. * Some of the parameter’s p-values are significant at <0.05. NCV: Nerve conduction velocity; YBI: Yoga-based intervention; ADA Ex. Regime: American Diabetes Association exercise regimen; FWCV: F-wave conduction velocity; IQR: Interquartile range; Pero.: Peroxidation

NCV parameters	Intervention	Intergroup change in mean ± SD (at baseline)	Intergroup change difference in mean ± SD (after six months)	p-value	Median	IQR
Right Peroneal Nerve Latency	YBI	4.94 ±1.94	0.724 ± 3.260	0.246	0.20	2.17
	ADA Ex. Regime	4.67 ±2.14	1.030 ± 3.135		0.91	1.6
Left Peroneal Nerve Latency	YBI	5.73 ±3.08	-0.445 ± 4.042	0.058	0.20	3.49
	ADA Ex. Regime	4.73 ±2.12	0.867 ± 2.779		0.90	1.4
Right Peroneal Nerve Amplitude	YBI	23.75 ±132.50	-21.105 ± 132.621	0.851	-0.05	2.75
	ADA Ex. Regime	22.80 ±120.80	-0.294 ± 2.056		-0.09	1.5
Left Peroneal Nerve Amplitude	YBI	31.27 ±145.51	-27.544 ± 146.044	0.273	0.30	3
	ADA Ex. Regime	28.85 ±141.68	-0.412 ± 1.709		-0.40	2.58
Right peroneal nerve NCV	YBI	42.48 ±9.37	0.306 ± 13.451	0.048	0.69	11.3
	ADA Ex. Regime	41.91 ±7.70	5.328 ± 13.423		7.10	16.6
Left Peroneal Nerve NCV	YBI	43.43 ±10.19	-2.663 ± 16.158	0.016	2.20	13.2
	ADA Ex. Regime	42.70 ±5.30	4.633 ± 10.058		5.60	12.34
Right Tibial Nerve Latency	YBI	5.91 ±2.53	0.576 ± 3.744	0.530	0.59	3.2
	ADA Ex. Regime	5.36 ±2.42	0.593 ± 2.748		0.80	2.64
Left Tibial Nerve Latency	YBI	6.24 ±2.99	0.204 ± 4.222	0.107	-015	3.4
	ADA Ex. Regime	5.53 ±1.71	1.010 ± 2.353		0.70	1.9
Right Tibial Nerve Amplitude	YBI	9.99 ±21.55	-3.472 ± 21.167	0.361	-0.20	4.4
	ADA Ex. Regime	6.44 ±3.46	-1.132 ± 4.118		-0.70	5.3
Left Tibial Nerve Amplitude	YBI	7.68 ±4.54	0.703 ± 5.991	0.435	0.30	7.29
	ADA Ex. Regime	7.28 ±3.79	-0.255 ± 3.001		-0.50	3.8
Right Tibial Nerve NCV	YBI	43.93 ±8.51	1.853 ± 11.170	0.769	3.99	15.9
	ADA Ex. Regime	44.81 ±12.58	2.451 ± 15.027		5.27	15.8
Left Tibial Nerve NCV	YBI	46.75 ±10.54	0.669 ± 14.399	0.494	0.50	13.88
	ADA Ex. Regime	44.46 ±13.61	2.262 ± 17.716		5.03	13.3
Pero. Right F-Latency (ms)	YBI	51.59 ±6.87	4.344 ± 9.257	0.014	5.40	13.24
	ADA Ex. Regime	49.26 ±5.68	9.338 ± 7.415		10.30	10.9
Pero. Left F-Latency (ms)	YBI	51.42 ±7.86	4.169 ± 10.043	0.036	4.60	12.9
	ADA Ex. Regime	49.58 ±5.92	8.744 ± 7.079		8.63	10.5
Pero. Right FWCV	YBI	38.40 ±4.93	-3.036 ± 7.950	0.336	-4.20	8.4
	ADA Ex. Regime	39.57 ±6.82	-4.958 ± 8.411		-4.98	9.8
Pero. Left FWCV	YBI	37.90 ±6.75	-1.614 ± 8.546	0.124	-2.70	10.3
	ADA Ex. Regime	39.47 ±7.58	-5.210 ± 8.257		-3.74	9.2
Tibial Right F-Latency	YBI	52.97 ±7.60	3.755 ± 10.830	0.209	3.23	12.6
	ADA Ex. Regime	51.23 ±7.12	7.319 ± 7.896		6.60	13.52
Tibial Left F-Latency	YBI	51.38 ±7.38	4.608 ± 9.864	0.219	4.99	13.5
	ADA Ex. Regime	51.40 ±6.39	7.657 ± 7.844		6.66	10.9
Tibial Right FWCV	YBI	38.90 ±6.02	-4.429 ± 8.005	0.994	-3.90	8.93
	ADA Ex. Regime	39.47 ±14.42	-5.793 ± 14.623		-3.70	9.62
Tibial Left FWCV	YBI	39.05 ±3.91	-3.702 ± 6.784	0.944	-3.95	6.25
	ADA Ex. Regime	38.20 ±6.72	-4.217 ± 6.465		-3.40	7.9

Impact of YBI and ADA Ex. Regime on biochemical parameters 

Table 5 illustrates the changes in biochemical laboratory parameters between the YBI and the ADA Ex. Regime groups in patients with diabetic neuropathy over a six-month period. Both intervention groups led to reductions in total cholesterol levels, with the YBI group showing a statistically significant decrease compared to the ADA Ex. Regime group. Although both groups exhibited reductions in LDL levels, no significant differences were observed in high-density lipoprotein (HDL) and low-density lipoprotein (LDL) levels between the two groups.

 The YBI group showed a significant increase in serum glutamic-oxaloacetic transaminase (SGOT) levels, whereas the ADA exercise regime group exhibited a decrease. Significant reductions in serum glutamic-pyruvic transaminase (SGPT) levels were observed in both groups, with the YBI group demonstrating a higher decrease. The YBI group showed a significant decrease in alkaline phosphate levels while slightly elevated in the ADA Ex. Regime group.

No significant differences were observed in blood urea and serum creatinine levels between the two groups. The YBI group exhibited a significant increase in urinary albumin levels, whereas the ADA Ex. Regime group showed a decrease. Both groups showed reductions in HbA1c levels, but the differences were not statistically significant. Both interventions reduced fasting and postprandial blood sugar levels, with the YBI group showing statistically significant decreases compared to the ADA Ex. Regime group.

The biochemical findings suggest that the YBI may have more beneficial effects on certain biochemical parameters, such as total cholesterol, alkaline phosphate, urinary albumin, and blood sugar levels, compared to the ADA Ex. Regime in patients with diabetic neuropathy.

**Table 5:** Impact of YBI and ADA Ex. Regime on biochemical parameters Non-Parametric Test: Comparison of two samples (Wilcoxon, Mann-Whitney test) compared the difference of post-data with pre-data in the YBI group and post-data with pre-data in the ADA Ex. Regime group. * Some of the parameter’s P values are significant at <0.05. YBI: Yoga-based intervention; ADA Ex. Regime: American Diabetes Association exercise regimen; HDL: High-density lipoprotein; LDL: Low-density lipoprotein; SGOT: Serum glutamic-oxaloacetic transaminase; SGPT: Serum glutamic-pyruvic transaminase; IQR: Interquartile range; FBS: Fasting blood sugar

Lab investigation parameters	Interventions	Intergroup change in mean ± SD (at baseline )	Intergroup change difference in mean ± SD (after six months)	P-value	Median	IQR
Total Cholesterol	YBI	188.62 ±44.95	-7.067 ± 30.691	0.048	-7.03	8
	ADA Ex. Regime	201.35 ±53.06	-12.610 ± 13.853		-10	15
HDL	YBI	44.20 ±10.53	-0.192 ± 6.209	0.548	1.25	5.22
	ADA Ex. Regime	37.70 ±10.88	-0.053 ± 3.570		1	5.07
LDL	YBI	122.48 ±54.36	-0.439 ± 28.746	0.233	-0.62	12.91
	ADA Ex. Regime	144.82 ±58.32	-2.626 ± 10.906		0.41	13.52
SGOT	YBI	27.84 ±13.12	1.375 ± 6.427	0.000	1.38	8.67
	ADA Ex. Regime	46.34 ±16.76	-4.318 ± 6.173		-4.20	0.95
SGPT	YBI	32.08 ±16.33	-0.711 ± 13.455	0.009	-0.71	10.47
	ADA Ex. Regime	44.80 ±14.20	-4.042 ± 7.607		-4.90	5.78
Alkaline Phosphate	YBI	116.90 ±31.46	-8.131 ± 27.219	0.017	-3.12	9.9
	ADA Ex. Regime	87.81 ±35.02	4.050 ± 14.255		-1.48	2.88
Blood Urea	YBI	27.28 ±7.82	-0.269 ± 4.094	0.788	-0.27	2.87
	ADA Ex. Regime	33.55 ±15.04	0.442 ± 4.544		0.37	3.67
S. Creatinine	YBI	0.89 ±0.35	-0.05 ± 0.241	0.310	-0.02	0.14
	ADA Ex. Regime	0.86 ±0.28	0.008 ± 0.092		0.02	0.10
Urinary Albumin	YBI	37.27 ±25.62	0.409 ± 6.395	0.003	0.41	2.23
	ADA Ex. Regime	40.48 ±10.02	-2.730 ± 1.995		-2.73	0
HbA1c	YBI	8.42 ±1.24	-0.846 ± 0.720	0.418	-0.70	0.95
	ADA Ex. Regime	8.47 ±1.42	-0.970 ± 0.812		-0.95	0.97
FBS	YBI	153.37 ±53.24	-7.190 ± 17.967	0.021	-3.90	18.75
	ADA Ex. Regime	164.83 ±47.64	-14.816 ± 31.284		-13.50	21.82
Post Prandial Blood Sugar	YBI	212.15 ±103.16	-23.544 ± 47.257	0.002	-9	24
	ADA Ex. Regime	254.07 ±72.43	-35.870 ±30.888		-30	37.25

Impact of YBI and ADA Ex. Regime on WHO-5

YBI exhibited superior improvement compared to the ADA Ex. Regime regarding WHO-5 well-being, with a significance level of p < 0.05. Table 6 illustrates the comparison of six-month changes in YBI and ADA exercise regimens on depression parameters in autonomic neuropathy among individuals with T2DM. The data did not display normal distribution, as indicated by the Shapiro-Wilk test (P < 0.05). Therefore, the Wilcoxon signed-rank test was utilized to compare 'post difference' values with 'pre Difference' values. Following the six-month intervention, intergroup changes were evident, with YBI showing greater improvement than the ADA Ex. Regime. However, no significant changes were observed in terms of PHQ-9 (refer to Table 6).

**Table 6:** YBI and ADA Ex. Regime on PHQ-9 health questionnaire in patients of diabetic neuropathy after six-month interventions Non-Parametric Test: Comparison of two samples (Wilcoxon, Mann-Whitney test) compared the difference of post data with pre-data in the YBI group and the difference of post data with pre-data in the ADA Ex. Regime group. * P value significant at <0.05. YBI: Yoga-based intervention; ADA Ex. Regime: American Diabetes Association exercise regimen; IQR: Interquartile range

Quality of life parameters	Intervention	Intergroup change in mean ± SD (at baseline)	Intergroup change difference in mean ± SD (after six months)	P-value	Median	IQR
PHQ-9	YBI	13.02 ±4.35	-2.944 ± 4.038	0.459	-3	7
	ADA Ex. Regime	15.22 ±5.21	-3.511 ± 4.104		-4.65	4.75
WHO-5 well being	YBI	41.10 ±8.78	26.143 ± 8.859	0.001	26	12
	ADA Ex. Regime	47.20 ±10.17	19.513 ± 7.775		19.32	8

This highlights that covariates such as participant age, diabetes duration, and HbA1c values were not significantly associated with autonomic function results (Table 7). The improvement in autonomic function was attributed solely to the intervention. Table 8 shows that covariates such as participant age, diabetes duration, and HbA1c values were not significantly linked to quality of life or depression status. The enhancement in both quality of life and depression status was solely attributed to the intervention.

**Table 7:** Assessment quality of life and depression Multiple linear regression analysis, Dependent variable-WHO Quality of Life, Adjusted R Square-0.007, P Value-0.318 Dependent Variable: PHQ at six months, Adjusted R Square-0.036, P Value-0.971 DM: Diabetes mellitus

Association of covariates with participant’s quality of life					
	Unstandardized Coefficients		Standardized Coefficients	T	Sig.
	B	Std. Error	Beta		
Age	-0.002	0.087	-0.002	-0.018	0.986
DM duration	.116	0.144	0.096	0.804	0.424
HbA1C	-1.368	0.802	-0.193	-1.707	0.092
Association of covariates with participant’s depression status					
Age	0.009	0.034	0.032	0.260	0.795
DM duration	-0.006	0.055	-0.014	-0.118	0.906
HbA1C	0.134	0.316	0.049	0.424	0.673

**Table 8:** Association of covariates with participant’s AFT Logistic regression analysis, Dependent variable-AFT, R square- 0.047 DM: Diabetes mellitus; AFT: Autonomic function test; SE: Standard error; Sig.: Significance; Exp (B): Exponentiation of the coefficient (B)

	B	SE	Wald	df	Sig.	Exp (B)
Age	0.015	0.031	0.244	1	0.622	1.015
DM duration	0.093	0.061	2.351	1	0.125	1.098
HbA1C	0.016	0.306	0.003	1	0.959	1.016

## Discussion

In this exploratory clinical trial, patients with already diagnosed DAN were selected and allocated into two groups: YBI and the ADA Ex. Regime. Standard care was common in both groups. Data analysis was done to compare the effectiveness of both interventions after intervening for six months on patients of both groups on selected parameters such as autonomic function, NCV, Biochemical parameters, and psychological parameters.

YBI reduced parasympathetic tone more than the ADA Ex. Regime. After six months, the YBI group's Valsalva Ratio (30:15) is higher than the ADA Ex. Regime group's p < 0.05. After a six-month intervention, the sympathetic activity indicator (SD2), as measured by YBI, showed a blower value than the ADA Ex. Regime, indicating a positive effect at p < 0.05. Compared to a YBI, the ADA exercise regimen significantly improved in the following areas: left peroneal NCV, right peroneal NCV, right peroneal F-latency, and left peroneal F-latency.

After sox-month interventions, ADA Ex. Regime showed significant decreases in total cholesterol, SGOT, SGPT, urinary albumin, fasting blood sugar (FBS), and PPBS parameters compared to YBI (p < 0.05). However, YBI outperformed ADA Ex. Regime in only one parameter, alkaline phosphate, with a significant difference (p < 0.05). Following a six-month engagement, when intergroup differences were seen, YBI outperformed ADA Ex. Regime regarding progress for WHO-5 wellness at p < 0.05. Following a six-month engagement regarding intergroup alterations, YBI outperformed the ADA Ex. Regime in terms of improvement regarding depression. It was noticed that co-variates such as participant's age, diabetes duration, and HbA1c values were not significantly associated with their autonomic function results, depression, and quality of life. The improvement in their autonomic function was clearly due to the intervention only.

The incidence of clinical autonomic dysfunction increases with both age and diabetes duration. Most diabetics develop diagnosable somatic and autonomic neuropathy after 15 years, according to laboratory studies. However, only a tiny number, between one in seven and one in ten, will develop symptomatic autonomic neuropathy [4]. Our study noted no significant association of autonomic neuropathy with the participant's age and diabetes duration.

Studies on cardiac patients have also yielded comparable positive results, including decreased blood pressure, cholesterol, and body weight [21,22]. Recent research has also shown that yoga can assist in managing diabetes. Yoga has been demonstrated to enhance glycemic control, lipid levels, and body composition (weight, BMI) in people with T2DM. [23] Cui et al.'s meta-analysis found a pooled weighted mean difference of -23.72 mg/dL (95% CI = -37.78, -9.65) for fasting blood glucose (FBG) and -0.47% (95% CI = -0.87, -0.07) for HbA1c [24]. In another meta-analysis, Kumar et al. revealed favorable benefits of yoga in contrast to conventional therapy alone for FBG (standardized mean difference (SMD) −1.40, 95%CI = −1.90, − 0.90) and HbA1c (SMD -0.64, 95%CI = −0.97, −0.30) [25]. However, this meta-analysis comprised trials with a short follow-up period (40 days). Since HbA1c indicates the average glycemia during the previous 8-12 weeks, a short follow-up period is insufficient to determine changes among yoga participants [26-29]. Furthermore, the authors solely investigated glycemic parameters. The authors did conduct a subgroup analysis based on the difference in intervention (i.e., breathing practice alone or a combination of asanas, breathing, and meditation). Still, no other intervention or sample factors were investigated as moderators of intervention impact. Finally, Vizcaino and Stover conducted a meta-analysis that included lipid profiles, blood pressure, and glycemic indices [28]. The authors showed substantial reductions in FBG for participants in the yoga condition compared to controls (mean difference = −25.72 mg/dL, 95% CI = −40.67, −10.76), but no significant changes in HbA1c or postprandial blood glucose. This meta-analysis did not account for baseline values in its studies, which might have influenced the results [28]. 

Yoga was also linked to substantial changes in lipid profile, blood pressure, BMI, waist/hip ratio, and cortisol levels. Overall, the studies fulfilled 41% of the methodological quality criteria, and the score was not linked with any outcome (Ps >0.05). Compared to a control condition, yoga reduced glycemic outcomes and other complication risk variables in persons with T2DM [29]. Another data analysis indicates that yoga can enhance the biochemical markers of blood glucose and lipid profiles in T2DM patients. As a result, yoga can be used as an effective and active supplemental therapy for T2DM. However, the study only examined yoga as a short-term therapy [30]. Poehlman et al.'s study adds to the body of evidence highlighting the positive effects of resistance and endurance training on insulin sensitivity. This research contributes to our understanding of exercise physiology and offers practical implications for designing comprehensive training programs that promote metabolic health in non-obese young women [31].

Yokoyama et al.'s work in diabetes research and clinical practice adds valuable evidence to the role of short-term, aerobic exercise in enhancing vascular health in individuals with T2DM. The findings underscore the importance of exercise as a modifiable factor in mitigating cardiovascular risk in this population [32].

Patients with diabetic neuropathy benefit from maintaining their blood pressure through moderate aerobic activity [33]. Long-term follow-ups are needed to assess its effectiveness for people with T2DM.

The study by Hegde et al. in diabetes care adds to the body of data supporting yoga's possible function in reducing oxidative stress in people with T2DM [8]. The three-month intervention makes it more practicable to include yoga in long-term diabetes care programs, emphasizing its overall health benefits beyond glucose control [34]. Chronic psychological stress has been associated with visceral obesity,T2DM, and their consequences. There was a positive relationship between the frequency of newly diagnosed illnesses and the number of stressful events [35]. Stress-induced changes cause elevated levels of hormones such as glucagon, cortisol, and growth hormone, which contribute to hyperglycemia. Hormones include catecholamines, prolactin, leptin, and neuropeptides. Prior systematic research described yoga's physiological and psychological impacts, concentrating on its capacity to improve glycemic control and insulin sensitivity, which decreases stress and depression while also improving quality of life [36].

Research has shown that yoga helps various symptoms, including physical functioning, depression, neurocognitive skills, and overall quality of life [37-40].

Studies on using yoga as a complementary and alternative medicine intervention have produced good physical, psychological, and quality-of-life results. Yoga is effective as an adjunct to antiemetic medication, in the treatment of menopausal symptoms, and in the reduction of acute and chronic cancer-related lethargy, diarrhea, and shortness of breath [41-43]. Significant psychological and quality-of-life results have included reductions in psychological distress, anxiety, and depression [41,43-45]. Two focus groups were held following a twice-weekly, 60-minute yoga intervention tailored for persons with diabetic peripheral neuropathy (DPN). The data revealed themes relating to improvements in bodily functions, notably neuromuscular movement-based and sensory functions, stress management, and sleep enhancement through breathwork, with social support playing an important role in the setting. These findings show that yoga can improve overall health in people with DPN by enhancing bodily functions, activities, involvement, and environmental variables. Yoga may be a strategy for improving overall well-being in people with DPN [46]. Findings can be generalized to T2DM patients with diagnosed autonomic neuropathy and aged between 35 to 60 years. The study can assist physicians and yoga practitioners learn how to manage diabetic neuropathy and depression via yoga practice, adding to the previous evidence that yoga has a favorable influence on participants' quality of life. In the future, robust randomized controlled trials (RCTs) with a bigger sample size and control group may be conducted to investigate the long-term effect of yoga on T2DM and diabetic neuropathy.

Limitations

This clinical trial was conducted in an open-label manner, and blinding was unfeasible due to the intervention's inherent characteristics. However, this trial was single-center and involved a small number of participants. Depression and quality of life assessments relied on subjective questionnaires. Since both groups received interventions, direct comparisons with a control group were not feasible, weakening the evidence's strength.

## Conclusions

In conclusion, the 24-week YBI in diabetic patients with peripheral neuropathy demonstrated significant improvements in neuropathy symptoms and stress levels. It effectively enhanced balance and muscle strength, surpassing the benefits of the ADA Ex. Regime in these aspects. Implementing YBI and ADA Ex. Regime with standard treatment may significantly improve patient outcomes. 

Considering the simplicity and affordability of yoga therapy, it emerges as a practical strategy to manage stress and complement existing treatments. Healthcare professionals should prioritize integrating yoga therapy into diabetic neuropathy management protocols. Further large-scale randomized trials are necessary to confirm these findings and solidify yoga therapy's role as a non-pharmacological treatment option.

## Appendices

Supplemental Material 1 

**Table 9:** YBI YBI: Yoga-based intervention

S. No.	Name of the practice (Asanas-Pranayamam)	Practice rounds	Timing of practice
1	Starting prayer: Asatoma Sat Gamaya	1 time	3 minutes
2	Asana		
	Preparatory Sukshma Vyayamas (Micro exercise) and Shithilikarana (relaxation) Practices (Neck Rotation, Hand clenching, Wrist Joint rotation, Elbow bending, Shoulder socket Rotation, Toe Bending, Ankle Bending, Ankle rotation, Knee bending, Full butterfly)	2 rounds each	30 minutes
	Tadasana (palm tree pose)	3 times each sides	
	Tiryak Tadasana (swaying palm tree pose)	3 times each sides	
	Katichakrasana (waist ratating pose)	3 times each sides	
	Bhujangasana (cobra pose)	3 times	
	Pashchimottanasana (back stretching pose)	3 times	
	Malasana (garland pose)	3 times	
	Mandukasana (frog pose)	3 times	
	Ardhaustrasana (half camel pose)	3 times	
	Ardhmatsyendrasana (half spinal twist pose)	3 times each sides	
	Gomukhasana (cow’s face pose)	3 times each sides	
	Makarasana (crocodile pose)	3 times	
3	Pranayama (Breathing exercise)		10 minutes
	Anulom (one nostril breathing)		
	Anulom-Vilom (alternate nostril breathing)	2 pranayama (breathing exercise) in each session for 5-5 min.	
	Naadi-shodhan (psychic network purification)		
	Ujjayi (the psychic breath)		
	Bharamari (bee breathing)		
4	Kriya and Mudra-Bandh		10 minutes
	Kapalbhati (frontal brain cleansing)	2 practices in each session for 5-5 min.	
	Agnisara (activating the digestive fire)		
	Ashwini Mudra (horse guesture)		
	Moolbandha (perineum contraction)		
5	Meditation		5 minutes
	Savita dhyan (rising sun meditation) (Meditation (for stress management for deep relaxation and silencing the mind))		
6	Resolve (I am completely healthy)		1 minute
7	Closing prayer: Sarvebhavantu Sukhinah	1 time	1 minute
	Total		60 minutes

Supplemental Material 2 

**Table 10:** Elements Used for Aerobic Training

S. No.	Name of (Asanas-Pranayamam)	Practice Round	Duration
1	Warm-Up	Slow jogging and stretching of major muscle groups	10 min
2	Aerobic Training	Brisk walking at a target heart rate of 50%–60% of heart rate reserve + resting heart rate rating of perceived exertion recumbent cycle	50 min
3	Cooling Down	Slow jogging and stretching of major muscle groups	5 min
Total Duration of Practice			1 hour

**Table 11:** ADA Ex. Regimes ADA Ex. Regime: American Diabetes Association exercise regimen

S. No.	Practice Type	Name of Practice	Practice Rounds	Practice Time
1.	Warm-up	Slow jogging and stretching of major muscle groups	2-3 times major joint movements	10 min
2.	Strength training	1 set of 5–20 repetitions of each exercise (5 repetitions will be increased each week if possible)		30–45 min
		- Wall push-ups	5-20 times	5-min
		- Back supported squats	5-20 times	5-min
		- Lunges	5-20 times	5-min
		- Heel raises with stretched hands	5-20 times	5-min
		- Prone trunk extensions	5-20 times	5-min
		- Straight leg raises	5-20 times	5-min
		- Chest lifts	5-20 times	5-min
		- Sit-ups	5-20 times	5-min
3.	Cooling down	Slow jogging and stretching of major muscle groups	2-3 times major joint movements	5 min
	Total duration of practice			60 minutes
